# Design and Implementation of 3D Animation Data Processing Development Platform Based on Artificial Intelligence

**DOI:** 10.1155/2022/1518331

**Published:** 2022-05-30

**Authors:** Quansheng Gao

**Affiliations:** College for Creative Studies, Changzhou Vocational Institute of Textile and Garment, Changzhou, Jiangsu 213164, China

## Abstract

Based on the whole process of computer-aided technology, a 3D animation data processing development platform based on artificial intelligence is designed and implemented. A random forest model for animation data processing and development is designed to mine the experience that can guide animation generation from the accumulated animation data. Based on the design goal and implementation principle of animation data processing and development platform, the attributes and categories of random forest model are abstracted. After standardizing a large number of historical data, the training sample set is obtained, and the random forest model is obtained after training. The parameters of the random forest model are continuously optimized by experiments, so that the learning model can better guide the dynamic animation data processing and development platform to generate animation to the satisfaction of users. The designed three-dimensional animation data processing and development platform interacts with the animation generation module, users, and system administrators. It can continuously receive the sample data of the animation generation module, automatically expand the number of training samples, analyze the status of the sample database, and put forward suggestions to the system administrator to update the learning model, so as to realize the initiative of learning. The experimental results show that the designed 3D animation data processing and development platform is effective and feasible.

## 1. Introduction

Three-dimensional animation technology [1–3] mainly relies on computer image technology, using three-dimensional model, light, material, and shooting of its change process to simulate the real picture of birth, and can display the shocking picture beyond reality through special effects [4–10]. The research of three-dimensional animation technology began in North America. After in-depth research on three-dimensional animation theory and computer animation system, three-dimensional computer animation system appeared in the 1970s and realized commercialization in the following 10 years. By the 1990s, 3D animation technology had made new breakthroughs in the field of film stunts, and 3D animated films became fashionable for a time [2].

Machine learning is the most popular artificial intelligence science at present [11–13]. It establishes a prediction model (training model) by learning the characteristics and results of known data sets (training samples). Using the model, the characteristics and results of unknown data can be predicted and measured. Classical machine learning algorithms include decision tree, neural network, support vector machine, etc.

At present, the traditional knowledge-based method faces two problems: (1) lack of learning ability, and a large amount of historical data is wasted; (2) the quality of finished animation can only depend on the level of the system designer and the user of the system—the user cannot participate in the generation of animation.

In view of this, this study builds a 3D animation data processing development platform based on machine learning algorithm to realize the learning ability of “continuous learning” of the platform. On the one hand, this platform solves the two inherent problems of the generation system. On the other hand, by building a circular learning system, the generation system has the ability of “continuous learning.”

## 2. Framework of Platform

### 2.1. Overall Architecture of the Platform

In order to solve the above two problems, the 3D animation data processing and development platform proposed in this article takes the user's evaluation of animation products and the historical data of the generation system as training samples and uses machine learning algorithm to generate a model with user evaluation as classification result to guide the generation system to generate animation that makes users more satisfied. However, users' evaluation of animation is a subjective judgment. Users' understanding of the text and users' psychological state and the color, music, action, and other factors of the animation itself will affect the evaluation results. In order to make the learning purpose more clear and the guidance more targeted, we need to choose a more specific evaluation object.

Scene is the background of animation. For users, scene is the most intuitive content of animation; For the generation system, the spatial attributes and layout attributes of the scene directly affect the subsequent model addition, color, lighting, and a series of planning. This study selects the animation scene as the evaluation object of users In addition, the selection of machine learning algorithm is a key problem in the implementation of 3D animation data processing and development platform. Among the machine learning methods, the method based on decision tree is widely used [14–19]. First, because the decision tree model is easy to understand, the problem-solving process can be intuitively understood through the working process of decision tree, and second because the decision tree can give accurate solutions to a wide range of problems. Random forest is an integrated learning device based on decision tree. It is known as “a method representing the level of integrated learning technology” because of its simplicity, easy implementation, low computational overhead, and strong performance in many real tasks.

### 2.2. Detailed Design of the Platform

The proposed 3D animation data processing and development platform is divided into two modules: generation system and learning system. Their design and implementation will be described below.

#### 2.2.1. Generation Module

**Figure 1:**
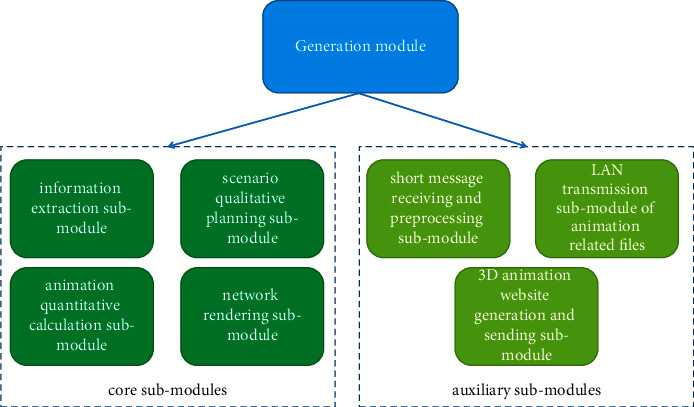
The generation module.

We build a sample database in generation module. The increase of sample data will stimulate the continuous updating of learning model, improve the guidance ability of learning system, and finally realize the ability of “continuous learning” of the system. The generation module (as shown in Figure 1) is divided into four core sub-modules: information extraction sub-module, scenario qualitative planning sub-module, animation quantitative calculation sub-module, and network rendering sub-module. Other auxiliary sub-modules are short message receiving and preprocessing sub-module, LAN transmission sub-module of animation related files, and 3D animation website generation and sending sub-module.

**Figure 2:**
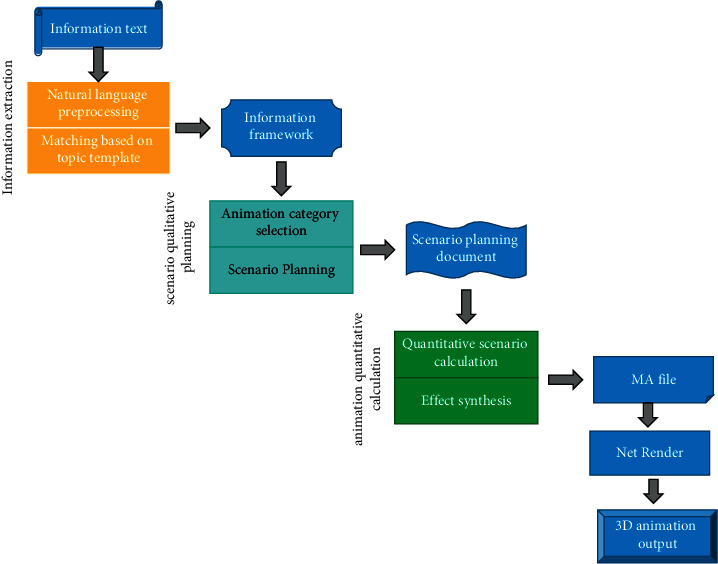
The flow chart of generation module.

The flow chart is shown in Figure 2.

The finished animation of the generation module depends on the animation material library. Animation material library mainly includes scene library, model library, background music library, etc. Both scene library and model library are Maya file libraries. The scene is the background scene where the animation takes place. A model is a physical model, such as a person, a flower, etc. First, the information extraction module extracts several topics and templates from the SMS text. Theme refers to the animation theme series extracted from SMS, such as birthday, playing basketball, new year, etc. Template is a concrete object, such as character template, location template, etc. For example, from the text message “I want to stay at home and don't want to play football,” the theme “play football” and the template “action” and “character” are extracted. These lines of information are stored in the IE file and become the basis for the qualitative planning of the plot. In the generation module, we use the software Protégé to build ontology library (animation scene library class, theme library class, template library class, background picture library class, scene space class, animation three-dimensional model class, etc.) and use SWRL language to write rules (including theme class rules, animation scene class rules, adding class rules used to produce variability effects on animation, deleting class rules, changing class rules, etc.), so as to realize the specific expression of different knowledge and the complex reasoning relationship between different data. The plot qualitative planning sub-module calls the corresponding ontology library and rule library according to the information framework; infers and completes the planning of scene selection, model increase, and decrease, action, color, deformation, and illumination; and writes the planning results into the plot planning document. The results of qualitative planning will be used as the basis of quantitative planning module. The quantitative module carries out quantitative calculation, operates the animation file, and then generates the animation scene file that can be rendered. Finally, we enter the network rendering module for rendering, generate playable animation files, and return them to the user.

The material selection of the generation module adheres to the principles of rationality, randomness, and diversity, that is the material conforms to the transmission content of short message text. The selection of material selection within a reasonable range is not interfered by people. Different animations will be obtained if the same short message text is input multiple times. The specific strategy of scene selection is as follows: first, several candidate scenes are obtained according to the theme, template atom, model, or special effect corresponding to template atom of information extraction. Then each candidate scene is scored according to the set scoring items (time, character, action, etc.), and the final total score is calculated. Finally, a candidate scene is randomly selected as the final scene. The higher the total score, the more likely the candidate scene is to be selected.

### 2.3. Learning Module

**Figure 3:**
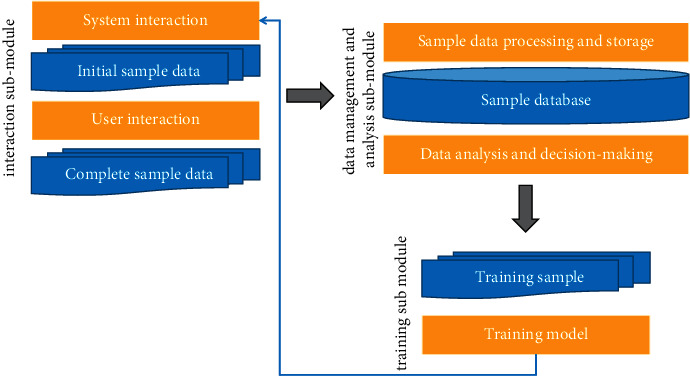
The flow chart of learning module.

The learning goal of the learning module is to help the generation system select more satisfactory scenes for users on the premise of adhering to the three principles of rationality, randomness, and diversity and finally improve users' satisfaction with animation. The learning module has three sub-modules: interaction sub-module, data management and analysis sub-module, and training sub-module. The flow chart is shown in Figure 3.

The interaction sub-module is responsible for interacting with the generation system and users. The historical data obtained through interaction with the generation module, including IE files with information framework, ADL documents with qualitative data (with selected scenes), finished animation products, and the user's evaluation of scene selection obtained through interaction with the user, are jointly used as training sample data. At the same time, the interaction sub-module should continuously transmit the learning results (model data) to the generation module to help the generation module make better choices. Interaction sub-module is an important guarantee to achieve learning objectives.

The data management and analysis sub-module realizes the unified storage, management, analysis, and decision of sample data. First, it receives the complete sample data transmitted by the interactive sub-module, extracts and processes the information, obtains the standard sample data, and stores it in the database, and then the sub-module analyzes and makes decisions on the data in the sample database.

The attribute list of the database is shown in Table 1.

As shown in Table 1, the difference attribute is used to select the SMS animation that needs interaction. Difference and IsUsed jointly decide whether to retrain. The strategies are as follows: (1) When the number of unused samples in the database exceeds the limit value, it is suggested that the system manager retrain the data; (2) when the number of samples whose difference value exceeds the limit value in the database, it is suggested that the system manager retrain the data; and (3) when the above two situations occur at the same time, the system manager is required to retrain the data. The training module uses training samples for training, and the obtained learning model is returned to the interaction module. The module accepts the judgment of the data management and analysis module to decide whether to train or not, so as to ensure that the learning model can be updated continuously with the operation of the generation system.

### 2.4. Random Forest Model for 3D Animation Data Processing

Random forest model [20–25] for 3D animation data processing is the key to realize the learning ability of animation data processing development platform. It can mine the experience that can guide animation generation from the accumulated animation data.

**Figure 4:**
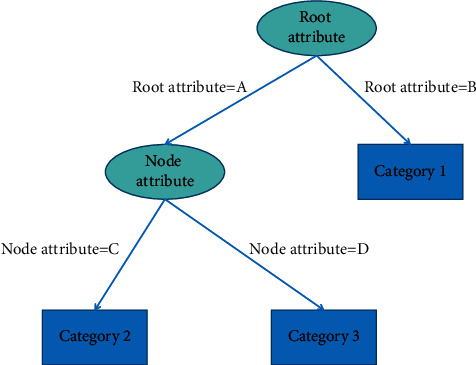
Decision tree.

Decision tree [18, 19] is a kind of inductive learning, and its structure is shown in Figure 4. The generation of decision tree is a recursive process. The key of decision tree learning is how to select the optimal partition attribute.

ID3 algorithm divides attributes based on information gain [23–25]. Assuming that the proportion of the *k*th sample in the current sample set *D* is *p*_*k*_, *k* = 1, 2,..., |*y*|, the definition of information entropy of *D* is shown as

(1) $$\begin{array}{c} \text{Ent}\left(D\right)=-\sum_{k=1}^{\left|y\right|}p_{k}\log_{2}p_{k}. \end{array}$$

Assuming that there are *V* possible values {*a*^1^, *a*^2^,...,*a*^*V*^} for discrete attribute *a*, and the sample with value *a*^*V*^ on attribute a is recorded as *D*^*V*^, the information gain obtained by dividing sample *D* by attribute *a* is as follows:

(2) $$\begin{array}{c} \text{Gain}\left(D,a\right)=\text{Ent}\left(D\right)-\sum_{v=1}^{V}\frac{\left|D^{V}\right|}{\left|D\right|}\text{Ent}\left(D^{V}\right). \end{array}$$

Bootstrap sampling is a sampling with return, that is we randomly take a sample from the initial data set (assuming that it contains *m* samples) and put it into the sampling set, then put the sample back into the data set, and continue to do this until the new sampling set has *m* samples. Random forest is a model composed of multiple decision tree classification models, which is sampled by bootstrap sampling. The basic idea of the algorithm is to use the bootstrap method to extract *t* sampling sets containing *m* samples, and then use the decision tree algorithm to train *t* decision tree models.

Different from the traditional decision tree algorithm, the random forest introduces the random attribute selection in the training process of the decision tree. The traditional decision tree directly selects the optimal attribute when selecting the partition attribute, while the base decision tree of the random forest first randomly selects an attribute subset and selects the optimal attribute from the subset. The classification results of random forest are determined by all set decision trees through voting. The commonly used voting methods are absolute majority voting method and relative majority voting method. Among them, the relative majority voting method refers to the random selection of one of the markers with the most votes.

Random forest can increase the difference between classification models by constructing different training sets, so as to improve the extrapolation prediction ability of combined classification models. Compared with decision tree, random forest has good advantages in solving over fitting problem. It should be noted that generalization error may occur within a certain limit.

## 3. Experiment

### 3.1. Experiment 1

Experiment 1 verifies whether the random forest model of the learning module in the 3D animation data processing and development platform can effectively learn users' preferences. Under specific text content, the animation scene with a user score of 1 is recorded as disgusting scene, and the animation scene with a user score of 5 is recorded as favorite scene. From the user's feedback on the animation, 18 text information animations with a score of 1 and 16 text information animations with a score of 5 are found; the corresponding text information, text content, and selected scene are extracted; and a comparative test on these 34 text information is conducted.

The specific operations are as follows: (1) Input the short message text into the generation module without learning module to get the scene selected by the system, and test the short message with the same content for 100 times; (2) input the text into the generation module guided by the learning module to get the scene selected by the system, and test the text with the same content for 100 times; and (3) compare the probability of disgusting scene/favorite scene between the two groups.

The comparison results are shown in Table 2.

### 3.2. Experiment 2

In the proposed 3D animation data processing and development platform, the main function of the learning module is to improve users' satisfaction with animation by helping the generation module select the scenes that users prefer. In Experiment 2, there were 50 experimental users—25 men and 25 women. The background information of experimental users is shown in Table 3.

We selected 500 animations from the original generation module. After the experimental user clearly understood the definition of the scene of the generation module, we scored the scene selection of these 500 animations, with an average score of 3.12. After the learning module was put into use, 100 animations were randomly selected to interact with experimental users to get score feedback. The average score was 3.71, an increase of 16.93%. The results show that the learning module can help the generation module to improve user satisfaction, and the growth rate has not increased significantly. The main reason is that users are not satisfied with the scene production in the material library of the generation system, resulting in no breakthrough in the highest score of user evaluation.

We use two experiments to verify the effectiveness of the learning module in the three-dimensional animation data processing and development platform, that is the learning module can learn users' preferences for the scene and help the generation module improve users' satisfaction with the finished animation. However, due to the objective limitations of other factors in the generation module, and in order to ensure the randomness and diversity of the animation, this improvement is within a certain range.

## 4. Conclusions

In this research, we mainly build a three-dimensional animation data processing and development platform, in which the design and implementation of generation module and learning module is the focus of this research. The learning module helps the generation module to continuously learn experience from historical data and generate animations that are more satisfactory to users. At the same time, the implementation of the learning module enables the generation module to have the ability of “continuous learning.” At present, the learning module mainly helps the generation module to select the scene. This learning mode can also be applied to the selection of model, character action ,and background music, so that the user's opinions can act on all aspects of the finished animation. The learning module is still in the improvement stage, and a large number of experiments are needed to find better system parameters, such as the number of base learners, so as to better serve the generation module.

Future research will be carried out from the following aspects. (1) Expansion of learning mode. A complete animation synthesis mainly includes three aspects: background scene, model, and music. The animation data processing and development platform designed in this article starts from the perspective of background animation scene selection. Whether this learning mode around background scene can be extended to the design and selection of model and music is a problem needs further research. (2) Optimization of parameters of animation data processing development platform. Animation data processing and development platform is a system involving many system parameters, such as the number of basic decision trees of random forest model and the setting of boundary values in data management and analysis. These parameters need further experiments and time test to demonstrate their effectiveness. (3) How to reduce the workload of users and system administrators. The proposed 3D animation data processing and development platform is currently in a semiautomatic state, and the system administrator needs to participate in the update of the learning model. Manual participation can ensure the stability and controllability of the system in the initial stage of system operation. With the maturity of platform functions, manual participation needs to be gradually reduced. The 3D animation data processing and development platform adopts the active learning method to obtain the user's evaluation marks for some text animation. At present, the interactive mode between the mobile platform and users is to show the text number to the user. The user accesses the platform and finds the text to be evaluated according to the number. The user evaluation is written into the sample database of the platform by the system administrator. In the next version of this platform, a more intuitive and convenient interactive interface for users can be built.

## Figures and Tables

**Table 1:** The attribute list of the database.

Number	Name	Meaning
1	ID	ID number
2	SMSN	SMS number
3	SSD	Standard sample data
4	IsUsed	Is this sample used as a trained sample
5	Difference	The distance value of the cluster closest to the sample
6	IsJudge	Is it rated by the user

**Table 2:** Comparative experiment of specific information text on the selection probability of user's favorite/dislike scene.

	User favorite sceneselection probability experiment			User aversion sceneselection probability experiment		
Growth rate of probability of learningmodule participating in favorite/disgustscene selection	0 to 10%	10% to 25%	25 to 40%	−10% to 0	−25% to −10%	−40% to −25%
Number	3	9	5	5	4	8

**Table 3:** The background information of experimental users.

Age	Occupation	Number of men	Number of women	Total number
15–25	Student	3	4	7
25–35	Graduate student	5	6	11
25–45	Workers	4	7	11
45–55	Workers	8	2	10
55–65	Unemployed and retirees	2	4	6
Above 65	Unemployed and retirees	3	2	5

## Data Availability

The data set can be accessed upon request.
